# Harmonic Vibration Analysis in a Simplified Model for Monitoring Transfemoral Implant Loosening

**DOI:** 10.3390/s24196453

**Published:** 2024-10-06

**Authors:** Qingsong Zhou, Louis Raymond Francis Rose, Peter Ebeling, Matthias Russ, Mark Fitzgerald, Wing Kong Chiu

**Affiliations:** 1 Department of Mechanical & Aerospace Engineering, Monash University, Clayton, VIC 3800, Australiawing.kong.chiu@monash.edu (W.K.C.); 2 Department of Medicine, School of Clinical Sciences, Monash University, Clayton, VIC 3800, Australia; peter.ebeling@monash.edu; 3 The Alfred Hospital, Melbourne, VIC 3004, Australiam.fitzgerald@alfred.org.au (M.F.); 4 National Trauma Research Institute, Melbourne, VIC 3004, Australia

**Keywords:** osseointegration implant loosening, structural health monitoring, bone–implant interface, vibrational analysis

## Abstract

A simplified axisymmetric model of a transfemoral osseointegration implant was used to investigate the influence of the contact condition at the bone–implant interface on the vibrational response. The experimental setup allowed the degree of implant tightness to be controlled using a circumferential compression device affixed to the bone. Diametrically placed sensors allowed torsional modes to be distinguished from flexural modes. The results showed that the structural resonant frequencies did not shift significantly with tightness levels. The first torsional mode of vibration was found to be particularly sensitive to interface loosening. Harmonics in the vibrational response became prominent when the amplitude of the applied torque increased beyond a critical level. The torque level at which the third harmonic begins to rise correlated with implant criticality, suggesting a potential strategy for early detection of implant loosening based on monitoring the amplitude of the third harmonic of the torsional mode.

## 1. Introduction

Osseointegration Prosthetic Limb (OPL) and Integral Leg Prosthesis (ILP) implants are treatments for lower limb amputees [1]. These implants are press-fit, hammered into bone remnants to facilitate the establishment of initial structural stability at the contact interface [2]. Subsequently, an external prosthetic device can be percutaneously connected to the distal extremity of the implants, enabling direct skeletal linkage with the prosthesis [3]. Compared to traditional socket prostheses, osseointegration implants can provide a higher quality of life due to their improved comfort (e.g., reduced skin irritation, pressure pain, etc.) and functionality (e.g., osseoperception, higher range of movements and activity levels, etc.) [4,5,6,7]. However, up to 3% of patients with transfemoral press-fit prostheses and 29% with transtibial press-fit prostheses may encounter implant loosening [4]. The occurrence of implant loosening necessitates surgical re-intervention, imposing discomfort and elevating the likelihood of subsequent repair surgeries [8].

Rigorous monitoring of the implant status becomes crucial for healthcare practitioners to plan early interventions and mitigate the risks associated with bone fractures and aseptic implant failures, particularly during load-bearing exercises [9,10]. Load-bearing exercises aim to stimulate bone ingrowth within the microporous layer of the implant [11]. However, excessive loading can induce considerable micro-movements at the bone–implant interface [12,13]. Micromotion of 30–200 μm [12,13,14] is considered to cause an immunoinflammatory reaction [15] triggered by wear particulate debris. This inflammatory response forms a low-elastic fibrous layer around the implant, thereby increasing the risk of aseptic loosening. Conventional medical imaging has a limited ability to detect implant loosening [16,17]. Radiological instrumentation scans may not detect any signs of implant loosening, even at a level of instability where a slight rotation of 1 mm could be excited by manual manipulation [8].

Vibration analysis has emerged as a promising non-radiative approach to detecting structural stability non-invasively. It has been successfully applied in biomechanics to detect implant osseointegration and monitor fractured femur healing [18]. The stability of bone–implant constructs can be examined by tracking shifts in resonant frequencies [19,20,21] in the dynamic responses. However, this approach was developed within the framework of linear vibration systems, assuming that the bone–implant system is well-fixed and the interface between the bone and implant remains bonded under mechanical excitation [22]. This assumption of linearity is not appropriate for characterising severe loosening status. Furthermore, resonant frequencies can be affected by many factors, such as mass loading, knee joint ligament, mode coupling, and bone atrophy [23,24,25]. Soft tissue may dampen higher-order vibration modes that are sensitive to bone healing [26,27,28]. These factors can alter the modal parameters, making it difficult to attribute a detected shift in resonant frequency to changes in interface conditions during early implant loosening.

An alternative approach based on harmonic distortions has been shown to improve the detection of loosening in total hip arthroplasty (THA) implants [29]. Applying sinusoidal excitation signals on the lateral femoral condyle and analysing the output acceleration signals at the greater trochanter showed the generation of harmonics in the output waveforms indicates loose implants. For example, a pioneering clinical study by Rosenstein, et al. [30], which applied sine wave excitation on the knees of patients and then analysed the output waves, was the first to demonstrate distorted output waveform and the presence of harmonics in the vibration responses of loose prostheses. Li, et al. [31] attempted to utilise similar techniques to differentiate various degrees of loosening: macroscopic loosening with free implant movement, early loosening with no macromovement, and secure implants. Their results demonstrated a distinct difference in the number of harmonics between firm and macroscopically loose implants. The effectiveness of such vibrometry methods was also investigated by Georgiou and Cunningham [17], who conducted a single-blind cohort study on 33 patients. They claimed that the vibrational method was 20% more accurate than radiographs clinically and did not require reference to baseline records. The occurrence of harmonics in vibration responses has been frequently reported in recent studies focused on the detection of knee implant loosening [32], transfemoral implant loosening [33], hip stem loosening [34], and acetabular cup loosening [35,36]. However, the mechanisms responsible for generating harmonics in loosening implants remain underexplored.

This study aims to investigate in more detail the mechanisms of harmonic generation in a press-fit implant model as a basis for the early detection of implant loosening. The experimental study employs a simplified model to investigate contact nonlinearity at the bone–implant interface, though it is not intended to faithfully replicate the in vivo mechanical properties of bone and implants. Instead, the objective is to control the tightness level at the interface and investigate the resulting vibrational response. Experimental modelling of implant interface control has been underexplored due to the complexity of human tissue. Cairns, et al. [37] proposed a simplified representation of interfacial fit using different thread configurations between the transfemoral implant and bone. In their experiment, implants were screwed into composite bones using various levels of insertion torque. However, they focused on the OPRA implant, which uses threading rather than press-fit to secure the implant to the bone. In our study, we used a hose clamp mechanism, which allows precise adjustment of the interface condition for press-fit implants.

This study presents vibration analyses of the contact construct with two tightness levels. Exciting the structure in its torsional mode activated the interfacial shear interaction. The harmonic amplitudes in the vibration response were investigated. We found that the experimental setup contains inherent asymmetry that influences the harmonic content in the vibration response. Therefore, the forced response of an axisymmetric finite element (FE) model was presented to help understand the relationship between interfacial slippage and spectral characteristics in the simplest scenario. The two-dimensional (2D) FE model examines the motion of a single cross-section taken from the experimental construct’s overlap region.

## 2. Experimental Investigation on Harmonic Generation in the Axisymmetric Model

### 2.1. Experimental Arrangements

**Figure 1:**
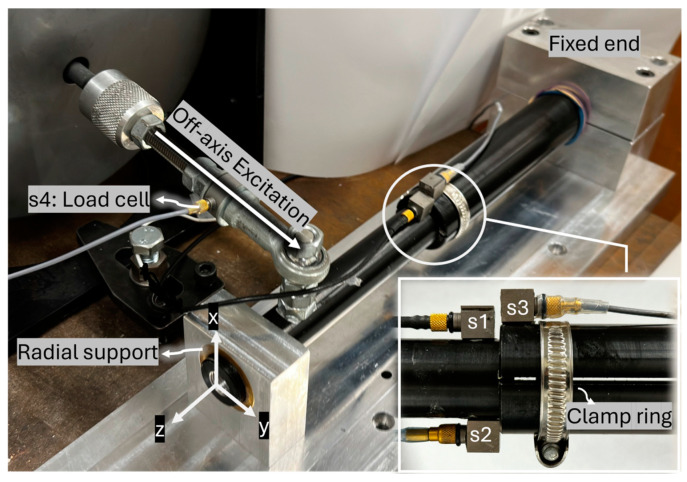
Experimental setup for the nonlinear vibration test.

a. Experimental setup:

The experimental setup is illustrated in Figure 1. An aluminium rod (implant) was inserted into a hollow cylinder (bone) with a 3 mm overlap length. The hollow cylinder represents the femur bone, and its proximal end is fixed into an abutment. A bush supported the distal end of the cylinder radially to constrain bending vibration while allowing twisting motion. An off-axis load provided the torque excitation, applied using a Bruel & Kjaer (B&K) electrodynamic shaker (Type 4824, valid frequency range: 0–5000 Hz) and a power amplifier (B&K 2732). Two slits were incorporated into the hollow cylinder to allow for the adjustment of insertion tightness (see Figure 1). This adjustment was made by turning the screw on a circumferential clamp near the contact area.

b. Data collection and processing method:

B&K 4507 accelerometers were employed in the experiment (frequency range: 0.3–6000 Hz, sensitivity: 10 mV/ms^−2^, measuring range: ±700 ms^−2^ peak). Two unidirectional accelerometers, denoted s1 and s2, were placed diametrically opposite on the implant near the contact region to record accelerations in the y-axis direction. Cross-spectrum analysis of the acceleration signals from s1 and s2 was conducted to evaluate the vibrational motion of the implant. The phase angle in the cross-spectrum analysis of s1 and s2 reveals the relation between the two sensors. A phase angle of 0 degrees means both accelerometers are moving in phase, indicating bending motion. Conversely, when the implant rotates about its longitudinal axis, the two sensors move in opposite directions, resulting in a phase angle of 180 degrees (out of phase).

A third accelerometer, labelled s3, was attached to the hollow cylinder to help examine interface slippage. The relation between s1 and s3 remains linear unless there is relative rotation between the bone and the implant. When the implant slides, the phase angle of s1 and s3 drifts from 0 degrees as they are no longer moving in phase. The auto-spectrum of accelerations was normalised by the magnitude of the first harmonic at the driving frequency, then the magnitude of the third harmonic after normalisation was investigated.

A load cell labelled s4 was used to determine the torque introduced by the shaker.

The data acquisition process, including time history data and spectral analyses of accelerations, was facilitated by the B&K PHOTON signal analyser with a sampling frequency of 31,920 Hz.

c. Experimental procedure:

The vibration behaviour of the bone–implant construct was investigated in the following procedure. First, two levels of press-fit were performed. The implant retained a certain degree of load capacity when subjected to the excitation provided by the shaker. The implant was subjected to broadband excitation from 300 Hz to 10,000 Hz to examine the resonant frequency. The placement of the accelerometers allowed for the isolation of the torsional modes.

The construct was excited at its torsional resonant frequency with different levels of input magnitude to study the generation of harmonics during the onset of slipping. The variation of normalised harmonic amplitude with increasing excitation level was investigated.

Lastly, additional experiments were performed to examine the effect of resonance on the spectral energy distribution among harmonics. The tightness of the implant was adjusted. The looseness was examined at various excitation frequencies (including one-half and one-third of the resonant frequency), such that the frequencies of higher-order harmonics would lie at the resonant frequency.

### 2.2. Experimental Results

**Figure 2:**
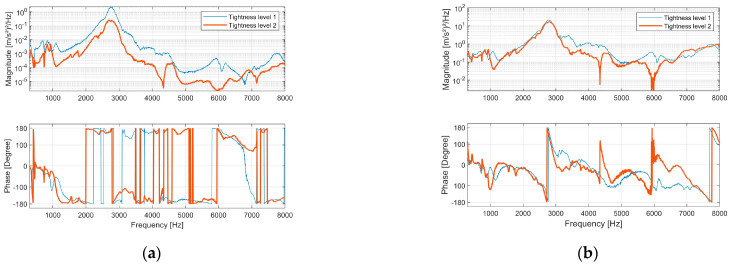
The implants were excited by a random signal from 300 Hz to 10,000 Hz. (**a**) Magnitude spectrum and phase spectrum of the cross power spectral density between acceleration s1 and s2. (**b**) Magnitude spectrum and phase spectrum of the frequency response function between acceleration s1 and s4.

The bone–implant structure was excited with broadband excitation from 300 Hz to 10,000 Hz. Figure 2a presents the cross-spectrum of s1 and s2. An out-of-phase pattern (180-degree phase angle) beyond 1100 Hz was evident, indicating rotational motion along the structure’s longitudinal axis. Below 500 Hz, s1 and s2 primarily exhibited in-phase behaviour, revealing that the vibration mode predominantly displayed abending motion.

Resonance was identified by a 180-degree phase shift in the frequency response function (FRF) of s1 and s4 (Figure 2b). This phase shift occurred at approximately 2760 Hz for both tightness-level models, corresponding to the most significant peak in the magnitude spectrum.

**Figure 3:**
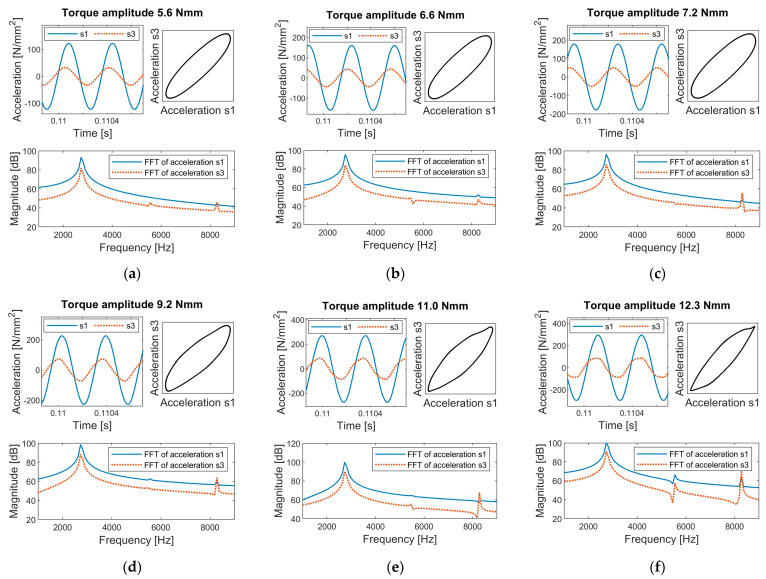
Time history and Fast Fourier Transform (FFT) of accelerations s1 and s3 for the experimental setup at tightness level 1, subject to excitation torques of (**a**) 5.6 Nmm, (**b**) 6.6 Nmm, (**c**) 7.2 Nmm, (**d**) 9.2 Nmm, (**e**) 11 Nmm, and (**f**) 12.3 Nmm.

The bone–implant construct was then excited at its resonant frequency of 2760 Hz with different excitation levels. The time history of the vibration response recorded by s1 and s3 for tightness level 1 is shown in Figure 3. The results demonstrate a clear association between interfacial slippage and the emergence of harmonics.

Before the onset of interfacial slippage, the time history of accelerations was purely sinusoidal. As the excitation torque increased, stick-slip interactions initiated at the bone–implant interface, leading to distortions in the acceleration peaks of s1 and s3. This distortion was evidenced by a noticeable difference in the harmonic content of the Fast Fourier Transform (FFT). As shown in Figure 3a,d, scenarios without slippage exhibited negligible harmonic amplitudes, whereas scenarios with slippage showed significant third harmonics in the frequency spectrum. At higher excitation levels, the distortions in the time history could be visually observed in the peak regions of the sinusoidal response (see Figure 3f), highlighting the strong nonlinear interaction between bone and implant.

The response was linear at the torque level of 5.6 Nmm (see Figure 3a). However, the accelerations were not in phase, and s3 led s1. The characteristic of resonance near natural frequencies can account for such a time lag or lead. In an undamped or slightly damped vibration system, the phase difference between the input and output signal can gradually change up to 180 degrees as the input frequency sweeps across its natural frequency [38]. Therefore, a phase lag or lead will be observed depending on the vibration characteristics and the driving frequency.

**Figure 4:**
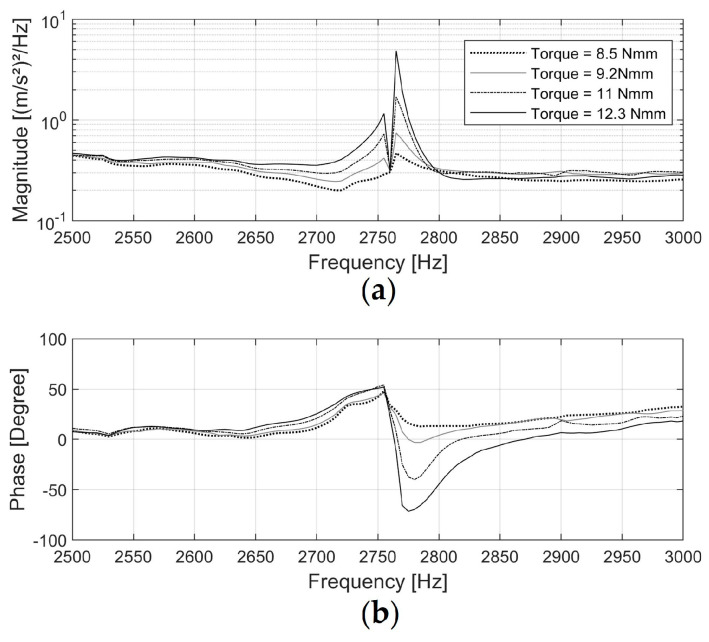
Magnitude spectrum (**a**) and phase spectrum (**b**) of the Frequency Response Function for acceleration s1 (excitation signal) and s3 (response signal) when the implant, tightened to level 1, was excited at its resonance of 2760 Hz.

Figure 4 shows the FRF of acceleration s1 (input signal) and acceleration s3 (output signal). The phase shift at 2760 Hz in the FRF between the two signals was approximately 50 degrees when driven at 8.5 Nmm, then increased to 120 degrees at 12.3 Nmm. This suggests that as the excitation amplitude increased, the natural frequency of the test specimen slightly shifted away from the driving frequency due to the change in interface status, causing a change in the time lead of s3. This input-level dependency of the frequency response is commonly observed in nonlinear vibration with hysteresis boundary conditions [39,40].

**Figure 5:**
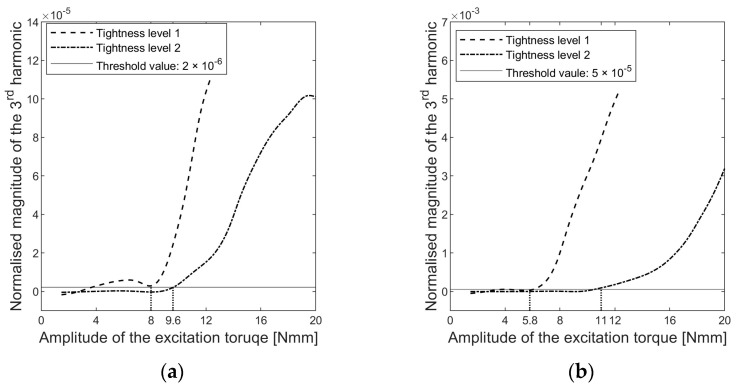
Changes in the magnitude of the third harmonics in the normalised auto-power spectrum density of (**a**) accelerations s1 and (**b**) accelerations s3.

The dependence of the third harmonic amplitude on the excitation torque level is shown in Figure 5. Thresholds were set to determine a critical torque level above which the harmonic amplitude increased significantly.

The critical torque level represented the load required to activate interfacial slip. However, the determination of the critical load differed for s1 and s3, with 8 Nmm and 5.8 Nmm for tightness level 1, respectively. Accelerometer s3 exhibited better sensitivity in diagnosing interfacial slippage compared to s1. The hollow cylinder is the passive part driven by the implant, and the time history of the vibration response suggested that the passive side of the contact pair generated more significant distortions for the same slippages, as shown in Figure 3. These distortions resulted in higher harmonic amplitudes at s3. As a result, sensor s3 captured implant sliding with greater accuracy than sensor s1 at the same sampling frequency.

**Figure 6:**
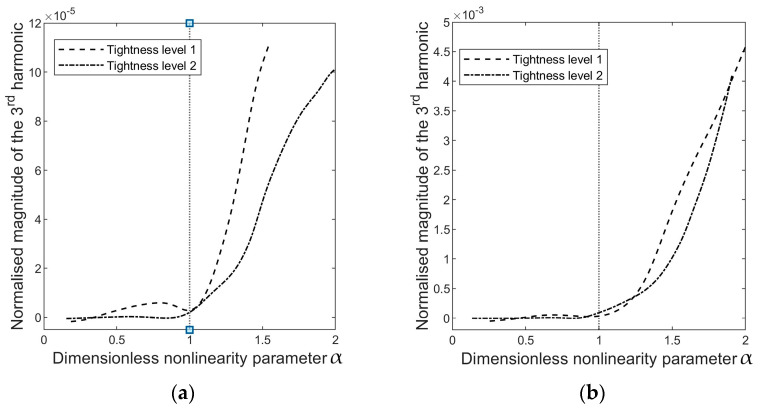
The magnitude of the third harmonic in the auto-spectrum of (**a**) accelerations s1 and (**b**) accelerations s3, as a function of *α* $=\frac{T_{E}}{T_{C}}$, where $T_{E}$ is the amplitude of the excitation torque and $T_{C}$ is the amplitude of the load capacity determined through a threshold value, as shown in Figure 5.

A dimensionless parameter *α* is defined to compare the energy dependency of harmonics,

(1) $$\alpha =\frac{T_{E}}{T_{C}}\;,$$

where $T_{E}$ is the amplitude of the excitation torque and $T_{C}$ is the amplitude of the critical load. Therefore, when *α* ≤ 1, the excitation level is insufficient to induce interfacial slippage, and no harmonics will appear in the cross-spectrum analysis. When *α* > 1, the interface status becomes nonlinear because it will shift between stick and frictional sliding. Figure 6 presents the evolution of the harmonics relative to *α*.

Nonlinearity arises when the interface slides, and the occurrence of the third harmonic manifests this nonlinearity. Therefore, it is important to emphasise that the emergence of harmonics in the vibration response is not a direct indication of implant tightness; rather, it measures the nonlinearity in the system, which depends on both the implant’s press-fit and the excitation level. The excitation amplitude at which the state of the system changes from linear (no harmonics) to nonlinear (harmonics arise) due to interfacial sliding is instrumental in quantifying the implant tightness. In other words, ‘tight’ or ‘loose’ describes the tightness of the implant relative to the excitation level. The implant is considered loose when interfacial slip initiates as α surpasses one. The excitation level required to induce implant slippage can quantify the implant criticality.

**Figure 7:**
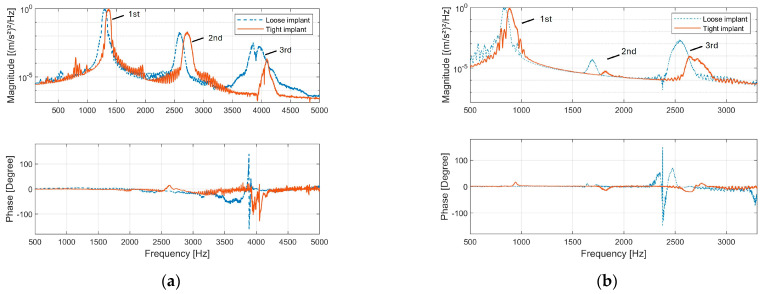
Magnitude and phase spectrum of the cross-power spectrum between acceleration s1 and s3: (**a**) The loose and tight implants were excited with the same voltage level at half (around 1400 Hz) the resonance. (**b**) The loose and tight implants were excited with the same voltage level at one third (around 700 Hz) of the resonance.

The tightness of the implant was re-adjusted and examined at half and one-third of the resonance frequency. The relatively tighter implant could be distinguished from the other implant according to the degree of phase shift in the cross-spectrum analysis of s1 and s3 (see Figure 7). The phase angle of s1 and s3 provides a straightforward indication of interfacial slippage. When slippage occurs, s1 and s3 fail to move in phase, and the phase angle between them drifts away from 0 degrees.

When comparing the second and third harmonics in the vibration response, it is observed that the spectral energy of higher-order harmonics is concentrated in the range of 2500 Hz to 2800 Hz, which corresponds to the resonance frequency band. This concentration indicates that resonance effectively limits the vibrational energy to its specific frequency range and thus influences the spectral energy distribution among harmonics. For instance, Figure 7b highlights a notable amplification of the third harmonic when the implant is excited at one-third of its resonance frequency. This observation suggests a potential strategy to enhance the detection of specific harmonics by selecting an optimal excitation frequency, thereby improving the sensitivity of implant monitoring.

**Figure 8:**
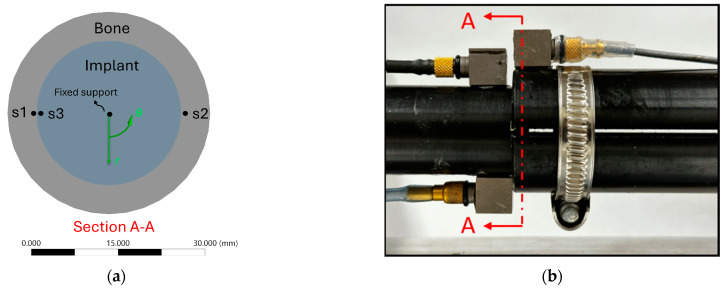
(**a**) The two-dimensional contact model which aims to simulate a representative cross section A–A of the contact region as shown in (**b**).

The experimental results show that interfacial slippages in the press-fit contact produce both odd and even harmonics in the vibration response (see Figure 3, FFT of accelerations and Figure 8b). However, in an axisymmetric model, friction energy dissipation should exclusively produce odd harmonics because the clipped nature of the acceleration signal due to friction energy dissipation indicates that its Fourier transformation only involves odd harmonics [41].

To understand the fundamental interplay between frictional energy dissipation and the resulting spectral characteristics, the following FE analysis employs a two-dimensional axisymmetric model with further simplification of torsional load. This analysis enables a direct probe of the shear stress at the interface, thus facilitating an investigation of harmonic generation under a simplified scenario of slippage.

## 3. Finite Element Analysis of Forced Response of an Axisymmetric Model

### 3.1. Finite Element Analysis (FEA) Setup

The 2D contact model is a sectional view perpendicular to the axis of symmetry of the contact region of the experimental construct, and it captures the behaviour of a single cross-section of the overlap region. The contact model (Figure 8) consists of a cross-sectional hollow cylinder with an inner diameter of 25 mm and a wall thickness of 5 mm, interfacing with the cross-section of a rod with a 25 mm diameter.

The interface was characterised as a press-fit frictional contact using Coulomb’s friction law, allowing slippage when the shear stress exceeds the limiting frictional stress. The contact pressure was configured at 0.3 Mpa and 0.35 Mpa. The friction coefficient was 0.4. The material properties of the cortical bone and titanium alloy were assigned for the hollow cylinder and rod material, respectively [12,13], as shown in Table 1.

The FEA was implemented in the commercial FE package (Ansys Transient Structural) to investigate the forced response of the press-fit contact model. The centre of the implant was fixed, and the contact side of the bone was driven harmonically by sinusoidal torques at its natural frequency (836 Hz, determined by Ansys Modal Analysis).

Accelerations in the circumferential direction were probed at two locations s1 and s3 (see Figure 8a). The data processing method is the same as in the experiment. The harmonic amplitudes were derived from the auto-spectrum of accelerometers at s1.

### 3.2. FEA Results

**Figure 9:**
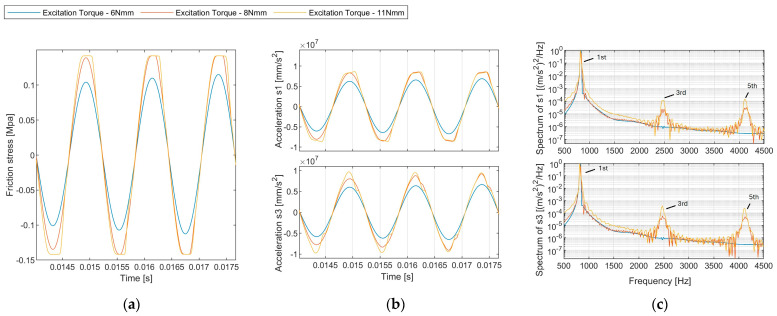
(**a**) Time history of the contact friction stress. (**b**) Time history of the acceleration probed at sensor s1 for different excitation levels (6 Nmm, 8 Nmm, 11 Nmm). (**c**) Normalised auto-power spectrum density of accelerations.

The response under varied excitation amplitude is presented in Figure 9. A noticeable distinction in harmonic amplitudes was evident between the scenarios with and without slippage. At 6 Nmm, where slippage was absent, the amplitude spectrum contained negligible harmonics. Elevated excitation levels at 8 Nmm and 11 Nmm led to prominent odd harmonics (third harmonic at 2445 Hz, fifth harmonic at 4075 Hz) in the amplitude spectrum.

**Figure 10:**
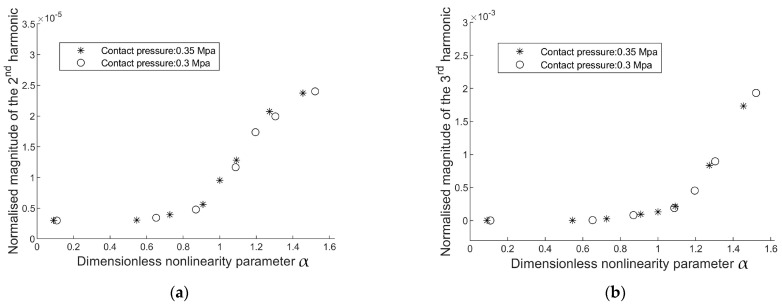
Evolution of (**a**) the second, and (**b**) the third harmonic amplitudes in the auto-power spectrum density of s1.

**Figure 11:**
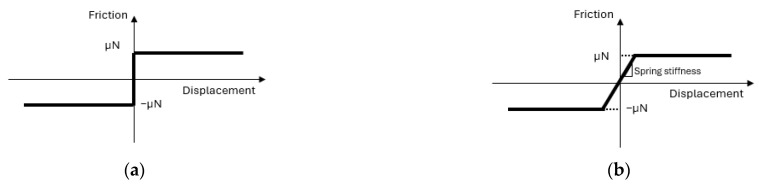
Interfacial contact models that are symmetrical with respect to relative displacement across the interface: (**a**) Coulomb friction model; (**b**) linear sliding contact model terminated by frictional sliding, where µ is the friction coefficient and N is the normal contact force.

The evolution of harmonics shows that the generation of second harmonics (magnitude scale: 2.5 × 10^−5^ is considerably smaller than that of the third harmonic (magnitude scale: 2 × 10^−3^). Hence, the magnitude of the second and third harmonics is not comparable, and only the third harmonics are related to frictional energy dissipation in the axisymmetric model (Figure 10). Different contact laws can influence harmonic generation in mechanical systems. However, only odd harmonics are predominantly generated in symmetrical contact laws (Figure 11), such as Coulomb’s friction law or spring contact law [13], because these contact laws lead to symmetric distortions of peaks in the sinusoidal response.

## 4. Discussion

The mechanism of higher harmonics generation was previously investigated in Contact Acoustic Nonlinearity. Biwa, et al. [42] investigated the transmission of a longitudinal normal incident wave passing through the contact interface of two blocks. They reported that the second harmonic decreased as contact normal pressure increased. Blanloeuil, et al. [43] conducted a numerical analysis on the nonlinear dynamics of a shear wave as it propagated through the frictional interface of two adjacent solids subjected to normal compression. They proposed that the nonlinearity of the frictional interface depended on both the magnitude of the time-harmonic incident wave and the characteristics of the friction model. Odd harmonics will be generated if the incident wave is reflected because its magnitude exceeds the maximum transmittable value [41]. These studies suggest that nonlinearities could be associated with the interface of bone and implant under mechanical excitation, such as switching between sticking–sliding or crack open–close actions. Klepka, et al. [44] applied vibro-acoustic excitation to investigate the nonlinearities of a cracked aluminium plate. They suggest that crack modes (i.e., open–close mode, slide mode, and tearing mode) are associated with the mode shapes of low-frequency excitation, revealing that interface behaviour can be manipulated by exciting it at the target vibration mode.

Building upon the insights from previous studies, we investigated the nonlinear behaviour in a bone–implant construct as the foundation for early implant loosening detection. Nonlinearity arises from the interactions between the bone and the implant at the contact interface. The vibration mode shapes, such as bending, torsional, and axial compression modes, determine the nature of these interactions. For instance, a “clapping” action is evident in the bending motion at the bone–implant interface, and frictional sliding can be observed when the implant rotates or is axially compressed. Clapping and friction-induced dissipative mechanisms may also occur at the interface when the implant undergoes coupled vibration modes, complicating the interpretation of the nonlinear response.

The experimental study characterised the interface between the bone and implant as a press-fit frictional contact. As loosening occurs, the contact pressure at the overlap decreases, reducing the implant’s stability. Our research has focused on the excitation of the torsional vibration mode, which has provided insightful revelations. This approach has facilitated a controlled investigation of shear stress interactions associated with torsional motion at the bone–implant interface. It is particularly effective for detecting changes in contact conditions as loosening progresses. The excitation level required to induce frictional sliding can quantify the implant criticality.

The evolution of harmonics magnitude under increasing excitation levels was investigated. The FEA results indicate that in an axisymmetric model, frictional energy dissipation in the press-fit contact exclusively produces odd harmonics. However, there is a discrepancy with the experimental findings where the amplitude of the second harmonic increases when the interface experiences slipping (refer to Figure 6). The second harmonic can be attributed to the inherent asymmetries in the experimental setup, specifically the off-axis excitation that causes a slight bending motion. As shown in Figure 2, the bending motion below 500 Hz is evident. Such asymmetries drift the acceleration response, resulting in uneven distortions of the positive and negative peaks in the sinusoidal acceleration response, ultimately influencing the observed harmonic content. Nevertheless, monitoring the amplitudes of odd harmonics in the vibration response is still instrumental in detecting slippage at the interface.

The results have significant implications for clinical implant monitoring. We suggest employing a two-sensor strategy to identify the torsional mode and excite the implant at its first torsional resonant frequency. The phase difference between acceleration s1 (placed on the implant) and acceleration s3 (placed on the bone) provides a straightforward indication of interface slippage. However, locating the sensor on the bone causes difficulty in bio-integration and powering [29]. Instead, sensors s1 and s2 (both on the implant) can be integrated into the extracorporeal part of the implant, thus facilitating non-invasive continuous monitoring. By only exciting the torsional vibration, the data acquired from sensors s1 and s2 can also provide information about interface slippage. However, this consistency is not guaranteed if the implant is excited at other vibration modes, particularly coupled modes, because the contact interaction moves beyond simple slip–stick behaviour.

In the experiment, the spatial distribution of the interfacial shear stress is assumed to be uniform in the short overlap. This assumption posits that the overlap slips simultaneously once the shear stress reaches the critical value. However, typical implant insertion lengths are long, ranging from 120 mm to 160 mm [3]. Therefore, when the distal end of the implant is excited by load, the spatial distribution of shear stress along the longitudinal direction becomes non-uniform [45,46], and the vibration mode shape will influence it. As the interface transfers load, the slippage zone will initiate at the stress-concentrating area and progressively propagate. Further investigation is required into the influence of this progressive slippage propagation on the vibration response probed extracorporeally at s1 and s2.

## 5. Conclusions

In this paper, an experiment was conducted to investigate the nonlinear vibration behaviour of an axisymmetric bone–implant construct with a press-fit contact. The press-fit level was controlled by adjusting a hose clamp encircling the bone. The bending vibration mode was differentiated from the torsional mode. Then, the implant was excited at the first torsional mode to induce shear stress interaction at the interface. A clear link between interfacial sliding and the nonlinearity of the bone–implant structure was demonstrated. The excitation amplitude at which the system transitions from a linear state (no harmonics) to a nonlinear state (harmonics present) due to interfacial sliding could provide a practical approach for quantifying implant tightness. A finite element analysis demonstrated that generating only odd harmonics in the response spectrum is directly related to a symmetrical interfacial contact law with respect to relative displacement across the interface. However, both even and odd harmonics were observed experimentally due to the asymmetry of the experimental setup caused by the eccentric load application. This study enhances our understanding of the nonlinear vibration characteristics in bone–implant systems, particularly emphasising the role of interfacial contact conditions. It highlights the fundamental relationship between stick–slip dynamics and the observed spectral characteristics and provides valuable insights for developing effective vibration-based techniques for the early detection of implant loosening.

## Figures and Tables

**Table 1:** Material properties for FEA.

	Density (kg/m^3^)	Poisson’s Ratio	Young’s Modulus (GPa)
Cortical bone	1829	0.3	17.6
Titanium alloy	4500	0.33	110

## Data Availability

The raw/processed data required to reproduce these findings cannot be shared at this time as the data also forms part of an ongoing study.
